# The genes expression difference between winged and wingless bird cherry-oat aphid *Rhopalosiphum padi* based on transcriptomic data

**DOI:** 10.1038/s41598-019-41348-1

**Published:** 2019-03-18

**Authors:** Rong-Jiao Zhang, Jing Chen, Li-Yun Jiang, Ge-Xia Qiao

**Affiliations:** 1 Key Laboratory of Zoological Systematics and Evolution, Institute of Zoology, Chinese Academy of Sciences, No. 1 Beichen West Road, Chaoyang District, Beijing, 100101 P.R. China; 2 College of Life Science, University of Chinese Academy of Sciences, No. 19, Yuquan Road, Shijingshan District, Beijing, 100049 P.R. China

## Abstract

Aphids produce wing and wingless morphs, depending on the environmental conditions during their complex life cycles. Wing and wingless variations play an important role in migration and host alternation, affecting the migration and host alternation processes. Several transcriptional studies have concentrated on aphids and sought to determine how an organism perceives environmental cues and responds in a plastic manner, but the underlying mechanisms have remained unclear. Therefore, to better understand the molecular mechanisms underlying the wing polyphenism of this fascinating phenomenon, we provide the first report concerning the wing development of aphids in bird cherry-oat aphid *Rhopalosiphum padi* with comparative transcriptional analysis of all the developmental stages by RNA-Seq. We identified several candidate genes related to biogenic amines and hormones that may be specifically involved in wing development. Moreover, we found that the third instar stage might be a critical stage for visibility of alternative morphs as well as changes in the expression of thirty-three genes associated with wing development. Several genes, i.e., *Wnt2*, *Fng*, *Uba1*, *Hh*, *Foxo*, *Dpp*, *Brk*, *Ap*, *Dll*, *Hth*, *Tsh*, *Nub*, *Scr*, *Antp*, *Ubx*, *Asc*, *Srf* and *Fl*, had different expression levels in different developmental stages and may play important roles in regulating wing polyphenism.

## Introduction

Organisms can flexibly alter their phenotypes in response to external stimuli to adapt to their surrounding environments, a phenomenon referred to as phenotypic plasticity^1^. In particular, some species can display different morphs of phenotypic plasticity despite having the same genotype in response to specific environments, a phenomenon referred to as polyphenism^2,3^. This is an adaptive behavioral strategy widely used by insects that live in heterogeneous environments, such as the wing and wingless morph development of aphids^4–6^.

Aphids are very important sap-sucking insects in Aphidoidea (Hemiptera), with more than 5000 known species in the world^7^. Aphids with complicated and diverse biotypes can reproduce wing or wingless morphs by distinct morphological differentiation in some species in response to environmental changes^8^. Wing and wingless variation is one of the normal characteristics to most aphid species; but wing morph variation is even more important to aphids with the complex and diverse life cycle, for example, the winged morphs of aphids are adapted for flight and migration to new locations, and the host-alternating species are wing-dimorphic, making it possible to alternate between primary hosts and secondary hosts to complete their life cycle. Serious pest challenges could then occur in any of the migration and host alternation processes. Therefore, wing and wingless variation is not only a trade-off strategy that has evolved in most insect orders for coping with complex and uncertain environments but also a key factor that leads to population expansion and infestation^9–11^.

Earlier studies have been based on morphological characters or transcriptional data and sought to determine how an organism perceives environmental cues and responds in a plastic manner, but the underlying mechanisms have remained unclear^10,12–17^. Utilizing scanning electron microscopy and histological sectioning, Ishikawa *et al*.^10^ observed and compared wing development in the winged and wingless individuals in *Acyrthosiphon pisum* and found the developmental processes underlying wing polyphenism^10^. Previous transcriptional studies have focused on some developmental stages of aphids, e.g., winged and wingless adults^12,14,16,17^, embryo and winged and wingless adults^15^, or fourth instar nymphs and winged and wingless adults^13^ and identified a handful of individual winged-associated genes, functional GO terms and pathways.

The bird cherry-oat aphid *Rhopalosiphum padi* (Linnaeus, 1758) is found in *Rhopalosiphum*, Aphidini (Aphididae: Aphidinae) and is distributed virtually worldwide, including in China, Japan, the Korean Peninsula, America, Canada, New Zealand, Russia, Egypt, Jordan, and Europe^18^. *R*. *padi* is holocyclic and anholocyclic, and is conservative in its choice of primary host, which includes *Armeniaca*, *Amygdalus*, *Malus*, *Pyrus* and *Prunus* plants. *R*. *padi* can alternate to secondary hosts, such as Cyperaceae, Iridaceae, Juncaceae, Typhaceae, dicots and Tuphaceae plants^18–20^. Therefore, *R*. *padi* is a typical wing-dimorphic aphid and its clonal populations, with the same genetic backgrounds, can be easily reared in the laboratory. Thus, *R*. *padi* is a useful experimental model for studying wing polyphenism.

The molecular mechanisms underlying the induction of winged and wingless aphids remain unknown. To better understand the gene regulatory basis of this fascinating phenomenon, we provide the first report concerning the wing development of *R*. *padi* with comparative transcriptional analysis of all developmental stages by RNA-Seq. Our results provide significant insights into the molecular mechanisms underlying how an organism relays environmental signals to its instars, increases our understanding of the critical stage that triggers alternative morph determination and provides a guide for monitoring the dynamics of migratory incidence in pests, predicting outbreaks of hazardous pests, and determining methods to control them.

## Results

### Offspring produced with winged morphs

Parthenogenetic females were subjected to crowded or solitary conditions, and we found that there were no obvious differences in external morphology in the first instar and second instar stage nymphs. In the third instar stage, small wing buds were identified in winged aphids, while there were no swollen structures in the thoracic part of the wingless aphids. Furthermore, during the fourth instar and adult stages, wing buds were enlarged and matured in winged aphids, while wing primordia completely disappeared in wingless aphids. In addition, we collected individuals for each instar aphid of the same genotype. Our analysis showed that 90% to 100% of offspring were winged when mothers were reared under crowded conditions for 16 h, whereas parthenogenetic females reared under solitary conditions produced less than 10% winged offspring (Table S1).

### RNA-Seq data analysis

The reference gene library was constructed by mixing equal quantities of total RNA from all samples, generating approximately 55.51 million total raw reads for the reference gene set. After filtering out the low-quality reads, we obtained 43.79 million clean reads. After filtering out repetitive and unannotated genes, the merged datasets yielded a total of 39,328 unigenes that were subjected to further analysis, and the total length, average length, N50, N90 and GC content of unigenes are 44,957,242 bp, 1,143 bp, 2,260 bp, 408 bp and 34.38%, respectively (Table S2).

In addition, 48 sample libraries of *R*. *padi* were generated, with an average of 22,886,759 raw sequencing reads and 22,741,712 clean reads after filtering low-quality reads (Table S3). After filtering, the clean reads were mapped to the reference gene library using the Bowtie2 tool^21^. The average mapping ratio with the reference genes was 94.79% (Table S4).

### Differentially expressed genes and pathways related to winged morph regulation

We compared the transcriptomes of all instar offspring at five sequential developmental stages based on gene expression results. Many differentially expressed genes and pathways were identified that may be involved in morphological divergence in aphid wing development.

We performed differentially expressed genes analysis and found that several biogenic amines and hormones may play important roles in regulating aspects of wing-morph development. More detailed inspection of the biogenic amines from RNA-Seq data revealed that these biogenic amine effects were mainly associated with serotonin, dopamine and octopamine, including Serotonin Transporter (SeT), Serotonin Receptor 1 (SeR1), Dopamine Transporter (DoT), Dopamine Receptor (DoR, DoR1, DoR2), Octopamine Receptor (OcR, OcR1, OcR2, OcR3). For hormones, such as juvenile hormone, ecdysone and insulin, these were associated with Juvenile Hormone Esterase (JHE), Juvenile Hormone Epoxide Hydrolase (JHEH), Juvenile Hormone Binding Protein (JHBP), Juvenile Hormone Acid Methyltransferase (JHAM), Ecdysone Receptor (EcR), Ecdysone-induced Protein (EcP74, EcP75, EcP78, EcP93), Insulin Receptor (InR), and Insulin Receptor Substrate (IRS).

Moreover, we found thirty-three differentially expressed genes: *Flightin* (*Fl*), *Wingless* (*Wg*), *Engrailed* (*En*), *Vestigial* (*Vg*), *Apterous* (*Ap*), *Hedgehog* (*Hh*), *Aristaless* (*Al*), *Serrate* (*Ser*), *Patched* (*Ptc*), *Brinker* (*Brk*), *Teashirt* (*Tsh*), *Nubbin* (*Nub*), *Fringe* (*Fng*), *Rhomboid* (*Rho*), *Ultrabithorax* (*Ubx*), *Homothorax* (*Hth*), *Antennapedia* (*Antp*), *Decapentaplegic* (*Dpp*), *Spalt*, *Notch*, *Wnt1*, *Wnt2*, *Wnt11*, *Wnt16*, *Distal-less* (*Dll*), *Cubitus interruptus* (*Ci*), *Optomotor*-*blind* (*Omb*), *Sex combs reduced* (*Scr*), *Forkhead box protein O* (*Foxo*), *Achaete*-*scute complex* (*Asc*), *Serum response factor* (*Srf*), *Cell division control protein* 42 (*Cdc42*), and *Ubiquitin*-*activating enzyme E1* (*Uba1*).

To further identify the functions of these genes, we found that the Wnt, Wingless (Wg), Notch, Hedgehog (Hh), Decapentaplegic (Dpp), Ecdysone, Insulin, and Epidermal growth factor receptor (EGF-R) signaling pathways were coordinately involved in aphid wing development. Wnt signaling pathway regulation included *Wnt1*, *Wnt2*, *Wnt11*, and *Wnt16*. Wg signaling pathway regulation included *Wg* and *Vg*. Notch signaling pathway regulation included *Notch*, *Ser*, and *Fng*. Hh signaling pathway regulation included *Hh*, *Ptc* and *Ci*. Dpp signaling pathway regulation included *Dpp*, *Brk*, *Omb* and *Spalt*. Ecdysone signaling pathway regulation included *EcR*, *EcP74*, *EcP75*, *EcP78*, and *EcP93*. Insulin signaling pathway regulation included *InR* and *IRS*. EGF-R signaling pathway regulation included *En*.

### Biogenic amine and hormone-associated gene expression profiling among offspring in crowded and solitary conditions

From the RNA-Seq data, we examined the expression levels of genes associated with the transport and reception of serotonin, dopamine and octopamine and the enzymatic functions, proteins or transport and the reception of juvenile hormone, ecdysone and insulin. We clustered these expression patterns across offspring based on the fragments per kilobases of transcripts per million mapped fragments (FPKM) expression results (Table S5) for crowded and solitary conditions using hierarchical clustering analysis by MeV4.9.0^22^.

**Figure 1:**
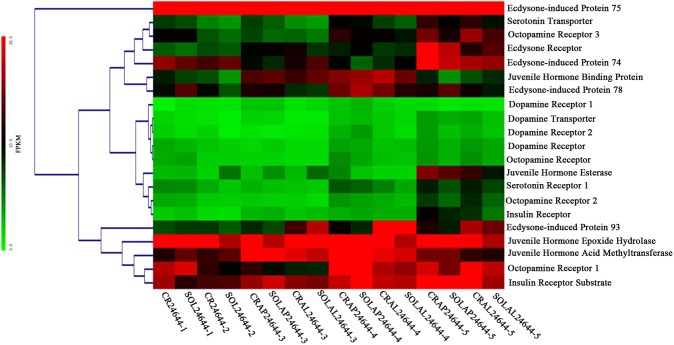
Heat map of biogenic amine and hormone-associated gene expression profiling among offspring in crowded and solitary conditions by RNA-seq. The FPKM represents the average of three replicates of gene expression levels in each instar aphids. The colored barcode gradients indicate the minimum value in green and the maximum value in red.

The results showed that biogenic amines and hormone-associated genes were grouped based on their expression profiles across all instar samples into 5 manually defined clusters (Fig. 1). The results were quite striking: the biogenic amines associated with serotonin, dopamine and octopamine exhibited similar expression patterns in crowded and solitary conditions. For example, serotonin associated with SeT and SeR1 exhibited a similar trend of expression: the first to third instars showed lower expression values, and the fourth instar to adult stages exhibited a gradually increased trend of higher expression. Dopamine related to DoT, DoR, DoR1, and DoR2 showed lower expression values for all instars. Octopamine showed a similar expression pattern of OcR, OcR1, OcR2, and OcR3, namely, the first instars, which showed a higher expression level, the second and third instars displayed a similar trend of lower expression levels, and the fourth instar to adult stages showed higher expression. Particularly, OcR1 and OcR3 displayed higher expression, while OcR and OcR2 exhibited lower expression.

Moreover, the hormones related to the juvenile hormone, ecdysone and insulin, also showed a similar trend of expression patterns in crowded and solitary conditions. For instance, juvenile hormones associated with JHE, JHEH, JHBP and JHAM, showed different trends of expression patterns. JHE displayed lower expression levels in the first to fourth instars, while adult stages displayed higher expression values. JHEH showed higher expression values for all samples. JHBP exhibited lower expression levels in the first, second and adult stages and exhibited a gradually increased trend of higher expression levels in the third instar to fourth instar. JHAM showed higher expression values for all the instars, especially in the third instar and fourth instar. Ecdysone exhibited a variety trends in terms of EcR, EcP74, EcP75, EcP78 and EcP93. EcR showed higher expression in the adult stages relative to other instars. EcP74 displayed higher expression levels in the first, second and adult stages and showed slightly lower expression levels in the third instar and fourth instar. In addition, EcP78 showed higher expression values in the fourth instar and adult stages than the others, and EcP93 exhibited higher expression in the third instar to adult winged instar, while EcP75 showed the highest expression values at all aphid stages. Additionally, insulin associated with InR exhibited higher expression levels in the adult stages relative to the other instars, while insulin related to IRS showed higher expression levels at all stages.

### Wing-associated gene expression profiling among offspring in crowded and solitary conditions

We examined the transcriptomes of whole-body tissues of aphids at five sequential developmental stages and obtained thirty-three differentially expressed wing-morph regulatory genes. Then, we clustered these thirty-three wing-associated gene expression patterns across all developmental stages based on the FPKM expression results (Table S6) for crowded and solitary conditions.

**Figure 2:**
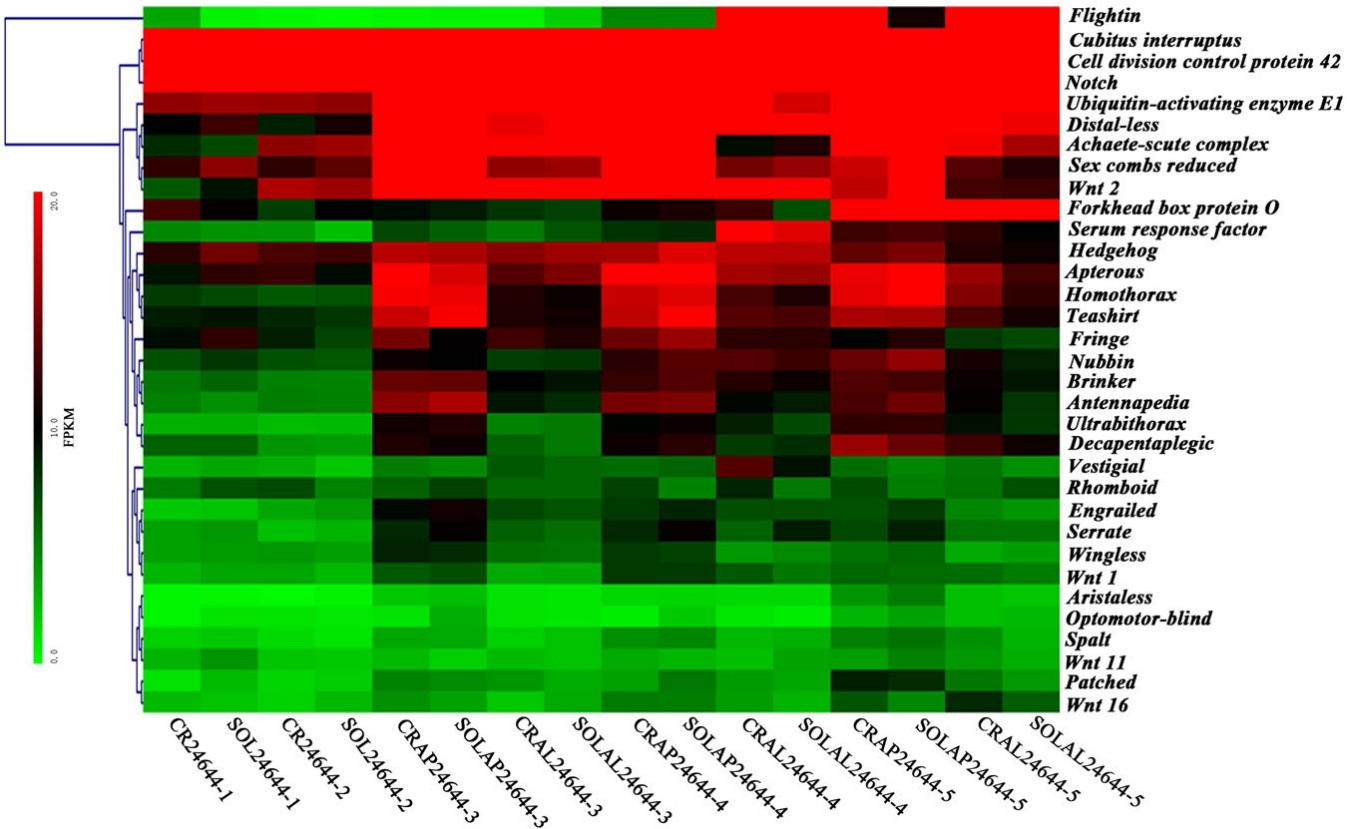
Heat map of wing-associated gene expression profiling among offspring in crowded and solitary conditions by RNA-seq. Genes were clustered via MEV using average FPKM expression values for each of the three replicates. Expression values are relative to one another within each gene, with red representing the highest and the green the lowest values.

The results showed that wing-associated genes were grouped by their expression profiles across all samples into seven manually defined clusters (Fig. 2). Upon further inspection of the heat map of the wing-associated gene expression profiling, we found that these genes exhibited a similar expression pattern in crowded and solitary conditions. For example, *Ci*, *Cdc42* and *Notch* cluster as one group and showed higher expression values at all stages. *uba1*, *Dll*, *Asc*, *Scr* and *Wnt2* were another group, *Foxo*, *Srf*, *Hh*, *Ap*, *Hth*, and *Tsh* were another group, and *Fng*, *Nub*, *Brk*, *Antp*, *Ubx* and *Dpp* formed an additional group. These three groups showed similar expression patterns: the expression in the first and second instars is almost the same, showing slightly lower expression levels; the third to adult stages exhibited higher expression values; and the first group showed higher expression levels relative to the rest of the groups. *Vg*, *Rho*, *En*, *Ser*, *Wg*, and *Wnt1* and *Al*, *Omb*, *Spalt*, *Ptc*, *Wnt11*, and *Wnt16* form the remaining two groups. They both showed a similar trend of expression, displaying lower expression values at all stages. Additionally, *Fl* exhibited a differentially expressed pattern compared to other genes, becoming a lone group, where the first to third instars showed lower expression values and the fourth instar and adult stages exhibited higher expression. Moreover, comparing the gene expression of apterae and alatae at the same stage of aphids, we found that most of the genes, i.e., *Ci*, *Cdc42*, *uba1*, *Dll*, *Asc*, *Scr*, *Wnt2*, *Foxo*, *Ap*, *Hth*, *Tsh*, *Fng*, *Brk*, *Antp*, *Ubx*, *Dpp*, *En*, *Ser*, *Wg*, *Wnt1*, *Al*, *Spalt* and *Ptc*, exhibited higher expression in apterae relative to alatae, while *Fl* exhibited higher expression levels in alatae than apterae. In addition, the rest genes, such as *Notch*, *Srf*, *Hh*, *Nub*, *Vg*, *Rho*, *Omb*, *Wnt11* and *Wnt16*, showed different expression values in terms of alatae and apterae.

## Discussion

Environmental factors can affect the wing polyphenism, crowded conditions were sufficient for high production of winged offspring by a group of aphids, while aphids reared under solitary treatment conditions never produced winged aphids or produced fewer winged offspring, indicating that crowded or solitary conditions result in drastic changes for aphids and are conditions that require gene expression responses. This result was consistent with *A*. *pisum* study^17^. Thus, although we treated the aphids equally, individuals probably did not receive the same level of wing-inducing signal during the 16 h of crowded or solitary stimulation. Despite equal treatment, the differential cue reception may be part of a stochastic polyphenism strategy, in which, once induced, aphids produce a stochastic output of winged and wingless morphs. This type of bet-hedging strategy is thought to be used in cases where environmental predictability is variable^23^, and it is possible that the relative gene expression changes in response to a biological property of the polyphenism.

Biogenic amines are known to play roles in regulating aspects of locust phase polyphenism^24^. Additionally, studies of insects, such as crickets, planthoppers and *A*. *pisum*, have revealed that alternative wing morphs develop in response to various environmental cues^2,8,25–29^, and responses to these cues may be mediated by hormones^8,9,27,30,31^. Therefore, we investigated whether biogenic amines and hormones were potentially involved in controlling *R*. *padi* wing polyphenism. In our study, biogenic amines and hormones showed a similar expression pattern in crowded and solitary conditions. For the biogenic amines, serotonin and octopamine showed varied expression levels in terms of different developmental stages, while dopamine showed lower expression levels for all instars. Biogenic amines associated with dopamine, serotonin and octopamine showed varied expression levels in different stages of aphids, indicating a possible functional role in wing phenotype determination through changes in the timing or expression level of transporters or receptors in different development stages in response to stressful environmental cues. Biogenic amines are small molecules synthesized from amino acids within neurons of the central nervous system. They are an integral part of the neuroendocrine system^32^. Biogenic amine signaling is of particular interest with respect to polyphenisms, as it plays a causal role in the phase polyphenism induced by crowding in locusts and other insects in response to stressful environmental cues^33–36^. Our results are consistent with previous studies, suggesting that transmission of environmental signals via biogenic amines is a possible factor in density-induced polyphenism and may be a common mechanism generally underlying aphid polyphenism.

Additionally, hormones related to juvenile hormone, ecdysone and insulin exhibited a varied trend of expression patterns at different stages of aphids, indicating that they may play roles in regulating aspects of wing polyphenism through changes in their timing or the expression of hormone enzymes, proteins, transporters or receptors in different development stages in response to environmental stimuli. Hormones are strong candidates for mediating the maternal response to crowding; hormone signals can traverse the hemolymph and affect the organism at the systemic level, and the polyphenism is marked by systemic differences between the morphs. Our results, combined with Braendle *et al*.’s study^9^, suggest that hormones possibly have an effect on aphid wing polyphenism. Combined with biogenic amines and hormone studies, we suggested a possible mechanism by which polyphenic aphids may have built upon density stress responses to influence alternative wing development.

To identify genes that could be involved in morphological divergence in aphid wing development, we examined the transcriptomes of all instars at five sequential developmental stages, and we found thirty-three differentially expressed genes. To further study, we found that these genes play well-known roles in anterior-posterior axis determination, dorsal-ventral axis formation, segmentation and wing hinge growth in insect wing development^37–47^ and likely perform wing-associated functions in aphids, potentially even playing roles in wing development. Therefore, we examined the expression levels of these wing-associated genes via hierarchical clustering at all developmental stages, and we obtained a preliminary heat map showing the timing and level of their expression. Most genes showed a similar trend in expression patterns. We observed a tendency of several genes, i.e., *uba1*, *Dll*, *Asc*, *Scr*, *Wnt2*, *Foxo*, *Srf*, *Hh*, *Ap*, *Hth*, *Tsh*, *Fng*, *Nub*, *Brk*, *Antp*, *Ubx* and *Dpp*, to be expressed at lower levels in the first and second instars but higher expression values during the third instar to adult stages. Another group of genes, including *Vg*, *Rho*, *En*, *Ser*, *Wg*, *Wnt1*, *Al*, *Omb*, *Spalt*, *Ptc*, *Wnt11* and *Wnt16*, showed lower expression values during all stages of aphids. A small number of genes, such as *Ci*, *Cdc42* and *Notch*, exhibited higher expression levels for all instars. Additionally, *Fl* showed a different expression pattern compared to the other genes, with lower expression values during the first to third instars and higher expression levels during the fourth instar and adult stages. According to the above gene expression results, we found that eighteen genes, i.e., *Wnt2*, *Fng*, *Uba1*, *Hh*, *Foxo*, *Dpp*, *Brk*, *Ap*, *Dll*, *Hth*, *Tsh*, *Nub*, *Scr*, *Antp*, *Ubx*, *Asc*, *Srf* and *Fl*, had varied expression values during different development stages, indicating that they may play roles in regulating aspects of wing polyphenism through changes in their timing or expression in certain development stages of the aphid.

Interestingly, comparing the gene expression patterns of apterae and alatae at the same life stages to further analysis these eighteen wing-associated genes, we found that most genes, such as *Wnt2*, *Fng*, *Uba1*, *Foxo*, *Dpp*, *Brk*, *Ap*, *Dll*, *Hth*, *Tsh*, *Scr*, *Antp*, *Ubx* and *Asc*, exhibited higher expression levels in apterae relative to alatae, although *Fl* exhibited higher expression levels in alatae than apterae. Additionally, a few genes, such as *Srf*, *Hh* and *Nub*, showed different expression values in terms of alatae and apterae. These genes have well-established functions in the wing development of insects, such as *Drosophila*, *Bombyx mori*, *Myzus persicae*, *M*. *crassicauda*, *Aphis gossypii* and *A*. *pisum*^12–17,37–48^. Together with previous studies, we discuss the functions of genes in regulating aspects of wing polyphenism in *R*. *padi* below.

To further study, we found that several genes were participated in signaling pathways, such as Wnt, Notch, Hh, Ecdysone, Insulin, and Dpp signaling pathways. These signaling pathways, which have previously identified functions in insect wing development^40,41,49–56^, are likely to play roles in aphid wing development. Several of the genes identified in this study are signal regulators, e.g., *Wnt2* is a member of the WNT gene family, which consists of structurally related genes that encode secreted signaling proteins involved in the Wnt signaling pathway, and studies of silkworms have shown that *Wnt2* plays a role in the regulation of wing development^53,54^. *Fng* is a boundary-specific signaling molecule that encodes secreted proteins involved in the Notch signaling pathway that are required for wing and margin formation, and *Fng* mediates interactions between dorsal and ventral cells during *Drosophila* wing development^49^. Additionally, *Uba1*, which is indirectly related to the Notch and Wnt signaling pathways, is known to catalyze the first step in ubiquitination, which is crucial for protein degradation^55,56^. A study in *Drosophila* proved that the loss of function of *Uba1* caused autonomous cell-cycle arrest^57^, and ubiquitination has been implicated in several signal transduction cascades, including the Notch and Wnt signaling pathways^58,59^. Thus, *Uba1* may be responsible for the temporal-specific activation of signaling pathways involved in wing-morph determination through ubiquitin-mediated control of cell proliferation. *Hh* is a signaling protein that participates in the Hh signaling pathway. It is specifically expressed in posterior compartment cells and plays a key role in patterning *Drosophila* imaginal discs^40,41^. *Foxo* is a subgroup of the Forkhead family of transcription factors that regulates the transcription of genes regulating diverse cellular processes^60^ as well as endocrine signaling proteins involved in the insulin signaling pathway. Studies of *M*. *crassicauda* proved that *Foxo* plays a role in wing-morph determination^15^. In addition, *Dpp* and *Brk* are signaling regulators of the Dpp signaling pathway. Studies of *Drosophila* have shown that the growth and patterning of the wing is controlled in part by the long-range organizing activities of *Dpp*^50–52^. *Dpp* is synthesized by cells that line the anterior side of the anterior or posterior compartment border of the wing imaginal disc. *Dpp* and *Brk* are expressed in different patterns and are thought to generate a concentration gradient that separates the anterior and posterior compartments to control wing development^61,62^. *Brk* encodes a protein that, as a *Dpp* target, negatively regulates Dpp signaling, and *Brk* expression is repressed by *Dpp*. In *Drosophila*, *Brk* can also repress the targets of a vertebrate homologue of *Dpp*, indicating that *Brk* underscores the importance of its negative role in regulating *Dpp* activity^63^. Thus, *Brk* may be involved in aphid wing determination.

Other identified genes are involved in major wing patterning events, e.g., dorsal-ventral (D-V) patterning genes, such as *Ap* and *Dll*^38,39,42^. Previous studies of *Drosophila*^38,64,65^ have shown that *Ap* is a member of the LIM family of developmental regulatory genes required for the normal development of the wing and haltere imaginal discs. Combined with research on pea aphids^14^, *Dll* is one of the wing-patterning genes that regulate wing development. Our results are consistent with previous studies, suggesting that *Ap* and *Dll* may play roles in differentiating the morphs. The wing hinge development genes, such as *Hth*, *Tsh*, and *Nub*^14^, were also identified; in *Drosophila*, *Hth*, *Tsh* and *Nub* function in the development of the wing hinge^43,45^. In addition, research on pea aphids^14^ has shown that *Hth*, *Tsh* and *Nub* are regulated genes participating in wing development in aphids. Additionally, Hox genes, i.e., *Scr*, *Antp*, and *Ubx*, also participate in wing patterning events^14,37,44^. In *Drosophila*, wings and halteres are the dorsal appendages of the second and third thoracic segments, respectively. In the third thoracic segment, wing development is suppressed by the homeotic selector gene *Ubx* to mediate haltere development^66^. Loss of *Ubx* function from developing haltere discs induces haltere-to-wing transformation, whereas ectopic expression of *Ubx* in developing wing discs leads to wing-to-haltere transformation^66–68^. Additionally, research on pea aphids^14^ suggested that *Scr* and *Antp* are regulated genes that participate in wing development, but their functions are not clear. Studies in *Drosophila* revealed that *Scr*, *Antp* and *Ubx* are homeotic genes in the thorax^37,39^; thus, they likely play roles in wing development.

The remaining identified genes, i.e., *Asc*, *Srf* and *Fl*, produce factors that regulate morphological features that likely play roles in regulating wing polyphenism. *Asc* encodes several homologous polypeptides that contain the BHLH (basic helix-loop-helix) domain, representing a subset of a complex gene family^69–71^. Four homologous genes were identified as *Asc* in silkworms^48^, expressing significantly higher levels in wing buds and indicating that *Asc* plays an important role in the formation of wing development. *Srf* is a member of the MADS-box family of transcription factors involved in orchestrating disparate programs of gene expression linked to muscle differentiation and cellular growth^72–74^. *Srf* encodes a MADS-box containing a transcriptional regulator, which is expressed in the intervein tissue of wing imaginal discs in *Drosophila*^75^, demonstrating that *Srf* plays a dual role during wing differentiation and acts in a dose-dependent manner to suppress the formation of wing veins. *Srf* is autonomously required in cells to promote the development of intervein cells. Previous studies on *Drosophila*, planthoppers and pea aphids^13,76–80^ suggest that *Fl* is a myosin rod-binding component of indirect flight muscles and is an essential part of the flight muscle contractile mechanism for thick filament assembly and sarcomere stability. In our study, *Fl* showed higher expression levels in wing morphs of fourth instar and adult stages, indicating that *Fl* likely plays an important role in the morphological divergence of the wing morphs.

Overall, through the timing and level of gene expression at all the developmental stages of aphids, we found that most genes showed a similar trend in the expression patterns, except for several genes that showed higher or lower expression values during all stages. For example, *uba1*, *Dll*, *Asc*, *Scr*, *Wnt2*, *Foxo*, *Srf*, *Hh*, *Ap*, *Hth*, *Tsh*, *Fng*, *Nub*, *Brk*, *Antp*, *Ubx* and *Dpp* were expressed at lower levels in the first and second instars but exhibited higher expression values in the third instar to adult stages. These results suggest that these genes may play roles in regulating aspects of wing polyphenism through changes in their timing or level of expression in certain stages of aphids. Moreover, we found that the strongest differences in gene expression in winged morphs occurred in the second instar and third instar nymphs, i.e., genes were expressed at lower levels in the second instar and exhibited higher expression values in the third instar, implying that the third instar is a critical stage for visibility of alternative morphs.

## Materials and Methods

### Insect rearing and sample preparation

A single apterous viviparous parthenogenetic female *R*. *padi* was collected from a laboratory at the Institute of Plant Protection, Chinese Academy of Agricultural Sciences, Beijing, China, in March of 2016. Samples colonies (voucher number 24644) generated from the above individual had been established in an incubator under long-day conditions (16 hours light: 8 hours dark, 22 °C) and a relative humidity of 65% on wheat seedlings for one year before being sacrificed in subsequent experiments. Samples from the same clonal colony preserved in 75% ethanol were maintained for slide-mounted voucher specimens for morphological identification. Species identification was performed by Ge-Xia Qiao and Li-Yun Jiang based on exterior morphological features of slide-mounted specimens. Prior to the start of all experiments, parthenogenetic females were maintained at a low density (one per plant) on wheat seedlings for ten generations to eliminate cross-generational effects on offspring morph determination^81^.

Environmental factors affecting the wing polyphenism during parthenogenetic generations are widely known to be influenced by density (contact stimuli), nutritional conditions (plant quality), temperature and photoperiod^9,81–83^. In this study, we used crowded and solitary conditions as wing-inducing stimulation. In the eleventh generation, the parthenogenetic females used for the following experiments were adults. The crowd-induced winged aphids were generated by placing twenty parthenogenetic females together in a square petri dish (length: 2 cm, width: 2 cm) without food. An equal number of parthenogenetic females were placed individually in a square petri dishes without food (one per square petri dish) for solitary treatment. Parthenogenetic females were subjected to crowded or solitary treatments for 16 h. Then, they were transferred to wheat seedlings (one per plant), allowed to produce nymphs for 24 h, and monitored for molting every day to collect all the developmental stages of the aphids.

**Figure 3:**
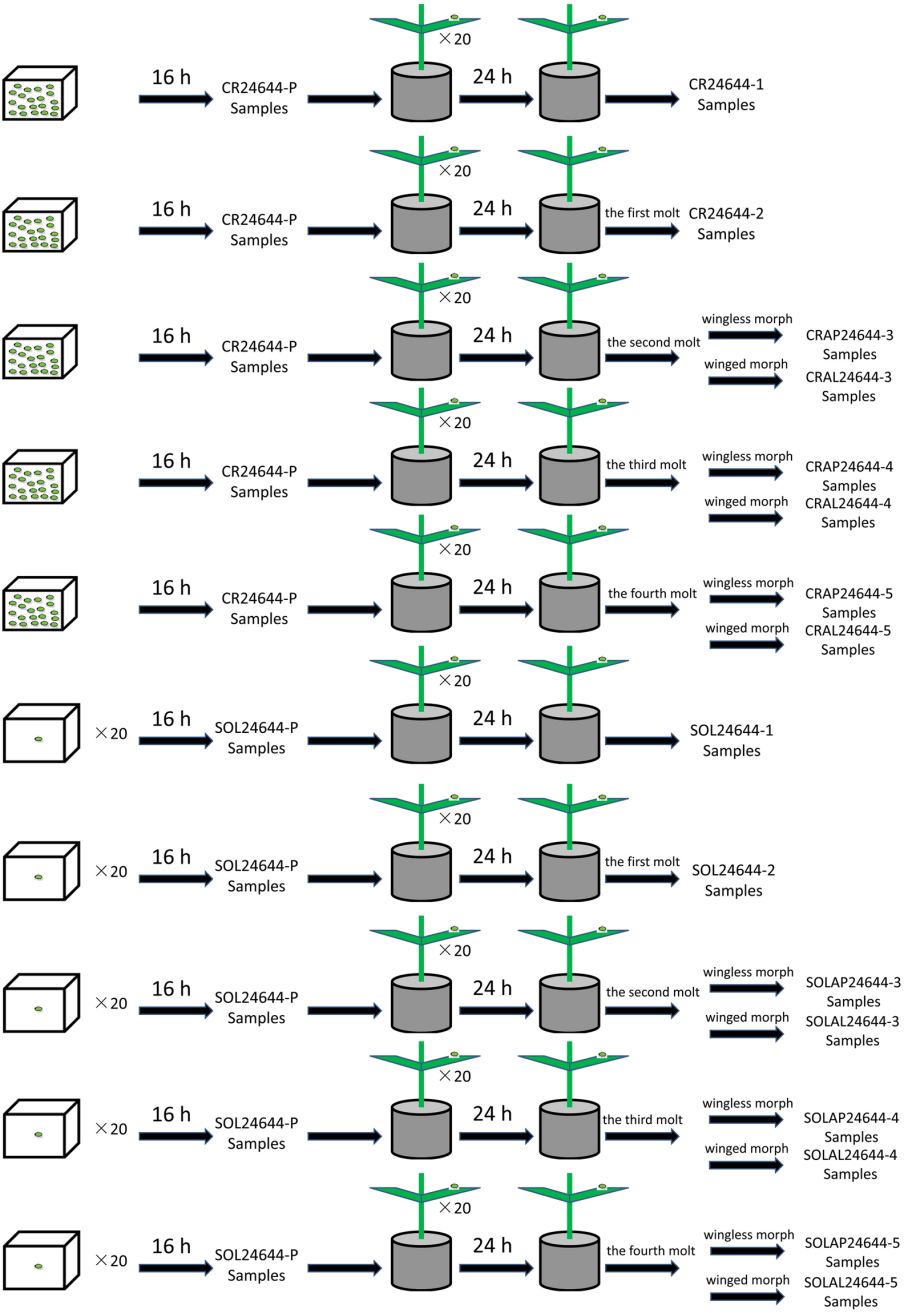
Aphid rearing and sample collection. As detailed in the methods, sixteen sets of aphids were used in this study. First, aphids were subjected to a solitary or crowded environment for 16 h respectively. Then, after 16 h of solitary or crowded environments, aphids were individually returned to wheat seedlings to feed and monitored for molting every day to collect all the developmental stages of aphids.

The first instar nymphs were directly collected from the crowded and solitary mother parthenogenetic females at 24 h, i.e., the “CR24644-1” and “SOL24644-1” samples. Similarly, the nymphs produced in the first 24 h were allowed to develop until the first molt, and the second instar nymphs were harvested, i.e., “CR24644-2” and “SOL24644-2” samples. Starting with the third instar nymphs, small wing buds were identified. For the fourth instar and adult stages, wing buds were outwardly visible. We collected the winged third instar and fourth instar and adult stage aphids from parthenogenetic females that experienced crowded conditions and produced more than 90% winged offspring, i.e., the “CRAL24644-3”, “CRAL24644-4” and “CRAL24644-5” samples. At the same time, a small amount of the third instar and fourth instar and adult stage aphids without visible wing buds were collected, i.e., the “CRAP24644-3”, “CRAP24644-4” and “CRAP24644-5” samples. We then collected the wingless third instar and fourth instar and adult stage aphids from parthenogenetic females reared under solitary conditions that produced more than 90% wingless offspring, i.e., the “SOLAP24644-3”, “SOLAP24644-4” and “SOLAP24644-5” samples. A small number of winged third instar and fourth instar and adult stage aphids were collected in the same manner, i.e., the “SOLAL24644-3”, “SOLAL24644-4” and “SOLAL24644-5” samples. All aphids were harvested during the same time of day to avoid any effects of photoperiod on the collection (Fig. 3).

In total, we collected individuals for all developmental stages of the offspring for crowded and solitary treatments (the first instar with 25 individuals, second instar with 12 individuals, third instar with eight individuals, fourth instar with six individuals and adult with five individuals), with three replicates for a total of 48 samples (Table S7). All samples (adult stages aphids were dissected to remove and discard ovaries with their developing embryos) were immediately frozen in liquid nitrogen and stored permanently at −80 °C.

### RNA extraction and library construction

Total RNA was isolated from pooled whole-body tissues of the first, second, third, fourth and adult stages aphids using an RNeasy mini Kit (Qiagen, Dusseldorf, Germany) according to the manufacturer’s instructions. Then, RNA was assessed for purity and integrity using an Agilent 2100 Bioanalyzer and an ABI StepOnePlus Real-Time PCR System (Agilent Technologies). cDNA libraries were sequenced in paired-end modes on an Illumina HiSeq 4000 system. A total of 48 libraries were constructed with three biological replicates per sample, and the reference gene library was constructed by mixing equal quantities of total RNA from all samples.

### De novo assembly and read mapping

The raw reads were obtained after sequencing and subsequently filtered to obtain clean reads. Then, clean reads were used in de novo assembly and read-mapping in comparison to the reference genes. The RNA-Seq data were de novo assembled using the SOAP program^84^, and sequences of the unigenes were produced by Trinity v2.0.6^85^. After that, unigenes from the reference gene library were further spliced and assembled to obtain non-redundant unigenes by Tgicl v2.0.6^86^. An additional set of 48 libraries was also filtered in the same manner using the SOAP program^84^ to obtain clean reads, then Bowtie2^21^ was used to map clean reads to the reference gene to obtain mapped reads.

### Functional annotation and gene expression

Functions of the unigenes were annotated by Blast v2.2.23^87^ with a cutoff E-value of 1E^−5^ to NCBI databases, including the Nr, Nt, Swissprot, Clusters of Orthologous Groups of proteins (COG) and Kyoto Encyclopedia of Genes and Genomes (KEGG) databases^88^. Additionally, based on the Nr annotation, Gene ontology (GO) classification was obtained by WEGO^89^ via GO IDs annotated by Blast2GO v2.5.0^90^. An alignment package, SOAP aligner (Version 2.20)^84^, was used to map reads back to the reference genes. Additionally, we mapped clean reads to the reference genes using Bowtie2^21^ in the first, second, third, fourth and adult stage aphids, and the relative expression levels of all the matched unigenes were normalized by transforming the clean data to FPKM^91,92^. Comparisons were made between all developmental stages of the offspring, and differentially expressed genes were screened by the NOISeq method^93^. Moreover, a fold change (FC) ≥ 2 and a probability ≥0.8 were used to filter significantly differentially expressed genes^94^.

## Supplementary information

Supplement information

## Data Availability

All data used in this manuscript are present in the manuscript and its supporting information. Additionally, the accession numbers of raw data will be available only after acceptance of the manuscript for publication.
