# Supplementary material for: The genes expression difference between winged and wingless bird cherry-oat aphid Rhopalosiphum padi based on transcriptomic data
Source: Sci Rep. 2019 Mar 18;9:4754. doi: 10.1038/s41598-019-41348-1 (PMC6426873; doi:10.1038/s41598-019-41348-1)
Supplement: Supplementary file 1 — Supplement information [file 41598_2019_41348_MOESM1_ESM.pdf]

The genes expression difference between winged and wingless bird cherry-oat aphid *Rhopalosiphum padi* based on transcriptomic data

Rong-Jiao Zhang[1,2], Jing Chen[1*], Li-Yun Jiang[1] & Ge-Xia Qiao[1,2*]

[1]Key Laboratory of Zoological Systematics and Evolution, Institute of Zoology, Chinese Academy of Sciences, No. 1 Beichen West Road, Chaoyang District, Beijing 100101, P.R. China. [2]College of Life Science, University of Chinese Academy of Sciences, No. 19, Yuquan Road, Shijingshan District, Beijing 100049, P.R. China.

*Corresponding authors.
Ge-Xia Qiao
Institute of Zoology
Chinese Academy of Sciences
Beijing, 100101
China
Tel/Fax: 0086-10-64807133/0086-10-64807099
E-mail address: qiaogx@ioz.ac.cn

Jing Chen
Institute of Zoology
Chinese Academy of Sciences
Beijing, 100101
China
Tel/Fax: 0086-10-64807257/0086-10-64807099
E-mail address: chenjing@ioz.ac.cn

Table S1. The winged and wingless offspring from individual females after 16 h of crowding or solitary treatments.

Sample name	Developmental stage	Rearing condition	Morph	Percentage of winged or wingless offspring (range)
CR24644-1	the first instar	crowded	wingless	100% wingless offspring
SOL24644-1	the first instar	solitary	wingless	100% wingless offspring
CR24644-2	the second instar	crowded	wingless	100% wingless offspring
SOL24644-2	the second instar	solitary	wingless	100% wingless offspring
CRAP24644-3	the third instar	crowded	wingless	0%–10% wingless offspring
SOLAP24644-3	the third instar	solitary	wingless	90%–100% wingless offspring
CRAL24644-3	the third instar	crowded	winged	90%–100% winged offspring
SOLAL24644-3	the third instar	solitary	winged	0%–10% winged offspring
CRAP24644-4	the fourth instar	crowded	wingless	0%–10% wingless offspring
SOLAP24644-4	the fourth instar	solitary	wingless	90%–100% wingless offspring
CRAL24644-4	the fourth instar	crowded	winged	90%–100% winged offspring
SOLAL24644-4	the fourth instar	solitary	winged	0%–10% winged offspring
CRAP24644-5	adult	crowded	wingless	0%–10% wingless offspring
SOLAP24644-5	adult	solitary	wingless	90%–100% wingless offspring
CRAL24644-5	adult	crowded	winged	90%–100% winged offspring
SOLAL24644-5	adult	solitary	winged	0%–10% winged offspring

Table S2. Assembly quality statistics of *R. padi* (the reference genes).

**Type**	**Total Number**	**Total Length (bp)**	**Mean Length (bp)**	**N50**	**N90**	**GC (%)**
Contig	53,310	51,342,361	963	2,067	325	34.40
Unigene	39,328	44,957,242	1,143	2,260	408	34.38

Table S3. Summarize the sequencing data for each sample.

Sample	Raw Data Size (bp)	Raw Reads Number	Clean Data Size (bp)	Clean Reads Number	Clean Data Rate (%)
CR24644-1F	1122991065	22918185	1119591935	22848815	99.69
CR24644-1S	1122984940	22918060	1105863899	22568651	98.47
CR24644-1T	1123027325	22918925	1105107094	22553206	98.4
CR24644-2F	1123024581	22918869	1102945214	22509086	98.21
CR24644-2S	1122961224	22917576	1103745139	22525411	98.28
CR24644-2T	1187098794	24226506	1162054502	23715398	97.89
CRAL24644-3F	1123006990	22918510	1118905347	22834803	99.63
CRAL24644-3S	1122966957	22917693	1119276522	22842378	99.67
CRAL24644-3T	1122958872	22917528	1119446993	22845857	99.68
CRAL24644-4F	1122993074	22918226	1120318556	22863644	99.76
CRAL24644-4S	1122997876	22918324	1120257551	22862399	99.75
CRAL24644-4T	1108930760	22631240	1105571075	22562675	99.69
CRAL24644-5F	1109634890	22645610	1104780705	22546545	99.56
CRAL24644-5S	1108466387	22621763	1104708969	22545081	99.66
CRAL24644-5T	1098663349	22421701	1096183949	22371101	99.77
CRAP24644-3F	1123006402	22918498	1120639996	22870204	99.78
CRAP24644-3S	1122958970	22917530	1120451150	22866350	99.77
CRAP24644-3T	1122978864	22917936	1119855310	22854190	99.72
CRAP24644-4F	1122968476	22917724	1117859246	22813454	99.54
CRAP24644-4S	1102483879	22499671	1099322497	22435153	99.71
CRAP24644-4T	1110003811	22653139	1105127380	22553620	99.56
CRAP24644-5F	1098574218	22419882	1095536610	22357890	99.72

CRAP24644-5S	1097538358	22398742	1094695525	22340725	99.74
CRAP24644-5T	1098606607	22420543	1095231683	22351667	99.69
SOL24644-1F	1123028746	22918954	1102770186	22505514	98.19
SOL24644-1S	1122982931	22918019	1091305264	22271536	97.17
SOL24644-1T	1122949366	22917334	1101866234	22487066	98.12
SOL24644-2F	1187148872	24227528	1168378687	23844463	98.41
SOL24644-2S	1123016594	22918706	1106291326	22577374	98.51
SOL24644-2T	1122916340	22916660	1100285200	22454800	97.98
SOLAL24644-3F	1122973474	22917826	1119855457	22854193	99.72
SOLAL24644-3S	1123022278	22918822	1120110453	22859397	99.74
SOLAL24644-3T	1122965340	22917660	1120385637	22865013	99.77
SOLAL24644-4F	1121014209	22877841	1116936723	22794627	99.63
SOLAL24644-4S	1122978423	22917927	1118736346	22831354	99.62
SOLAL24644-4T	1123017672	22918728	1118728114	22831186	99.61
SOLAL24644-5F	1100590176	22461024	1096308213	22373637	99.61
SOLAL24644-5S	1123016349	22918701	1119059795	22837955	99.64
SOLAL24644-5T	1196050500	23921010	1189604400	23792088	99.46
SOLAP24644-3F	1122976414	22917886	1119160000	22840000	99.66
SOLAP24644-3S	1123004687	22918463	1119476491	22846459	99.68
SOLAP24644-3T	1122986459	22918091	1118416523	22824827	99.59
SOLAP24644-4F	1109571925	22644325	1106372176	22579024	99.71
SOLAP24644-4S	1101312387	22475763	1098343134	22415166	99.73
SOLAP24644-4T	1100468999	22458551	1097407185	22396065	99.72
SOLAP24644-5F	1097037088	22388512	1093580089	22317961	99.68
SOLAP24644-5S	1095384661	22354789	1090085409	22246641	99.51
SOLAP24644-5T	1122945887	22917263	1118020946	22816754	99.56

Table S4. Alignment statistics of reads align to reference gene.

Sample	Total Reads	Total Mapped Reads (%)	Unique Match (%)	Multi-position Match(%)	Total Unmapped Reads (%)
CR24644-1F	22848815	95.13	74.4	20.74	4.87
CR24644-1S	22568651	94.16	72.48	21.68	5.84
CR24644-1T	22553206	94.06	72.57	21.49	5.94
CR24644-2F	22509086	93.8	72.73	21.07	6.2
CR24644-2S	22525411	94.12	73.11	21.01	5.88
CR24644-2T	23715398	93.28	72.27	21	6.72
CRAL24644-3F	22834803	94.34	72.7	21.64	5.66
CRAL24644-3S	22842378	94.78	73.47	21.31	5.22
CRAL24644-3T	22845857	94.57	72.94	21.63	5.43
CRAL24644-4F	22863644	95.12	72.31	22.81	4.88
CRAL24644-4S	22862399	94.81	71.87	22.94	5.19
CRAL24644-4T	22562675	95.41	72.31	23.1	4.59
CRAL24644-5F	22546545	95.95	72.32	23.63	4.05
CRAL24644-5S	22545081	95.47	71.27	24.2	4.53
CRAL24644-5T	22371101	95.35	70.58	24.77	4.65
CRAP24644-3F	22870204	94.55	72.29	22.26	5.45
CRAP24644-3S	22866350	95.02	73.27	21.75	4.98
CRAP24644-3T	22854190	94.43	71.49	22.94	5.57
CRAP24644-4F	22813454	94.07	70.18	23.89	5.93
CRAP24644-4S	22435153	95.3	73.3	22	4.7
CRAP24644-4T	22553620	93.88	69.96	23.93	6.12
CRAP24644-5F	22357890	95.33	70.61	24.72	4.67

CRAP24644-5S	22340725	95.42	70.54	24.88	4.58
CRAP24644-5T	22351667	95.43	71.99	23.44	4.57
SOL24644-1F	22505514	93.1	70.99	22.11	6.9
SOL24644-1S	22271536	94.06	72.09	21.97	5.94
SOL24644-1T	22487066	94.29	72.63	21.66	5.71
SOL24644-2F	23844463	94.18	73.4	20.79	5.82
SOL24644-2S	22577374	94.46	73.54	20.92	5.54
SOL24644-2T	22454800	94.84	74.12	20.71	5.16
SOLAL24644-3F	22854193	94.88	72.84	22.04	5.12
SOLAL24644-3S	22859397	94.76	73.12	21.64	5.24
SOLAL24644-3T	22865013	95.28	73.98	21.3	4.72
SOLAL24644-4F	22794627	94.21	71.2	23.01	5.79
SOLAL24644-4S	22831354	94.69	70.9	23.8	5.31
SOLAL24644-4T	22831186	94.65	70.81	23.83	5.35
SOLAL24644-5F	22373637	95.14	71.26	23.88	4.86
SOLAL24644-5S	22837955	95.31	70.83	24.48	4.69
SOLAL24644-5T	23792088	95.3	71.48	23.82	4.7
SOLAP24644-3F	22840000	94.85	72.03	22.83	5.15
SOLAP24644-3S	22846459	95.33	73.23	22.1	4.67
SOLAP24644-3T	22824827	95.35	73.43	21.92	4.65
SOLAP24644-4F	22579024	94.59	71.53	23.06	5.41
SOLAP24644-4S	22415166	94.73	71.28	23.46	5.27
SOLAP24644-4T	22396065	95.28	72.37	22.9	4.72
SOLAP24644-5F	22317961	95.35	71.38	23.97	4.65
SOLAP24644-5S	22246641	95.26	71.6	23.66	4.74
SOLAP24644-5T	22816754	95.24	72.3	22.94	4.76

Table S5. FPKM of biogenic amines and hormone-associated genes among offspring in crowded and solitary conditions.

Gene ID	Annotation Gene	CR24644-1	SOL24644-1	CR24644-2	SOL24644-2	CRAP24644-3	SOLAP24644-3	CRAL24644-3	SOLAL24644-3	CRAP24644-4	SOLAP24644-4	CRAL24644-4	SOLAL24644-4	CRAP24644-5	SOLAP24644-5	CRAL24644-5	SOLAL24644-5
Unigene2126_CR24644-1S	**SeT**	7.73	7.02	4.94	4.15	7.53	6.38	4.44	3.9	9.86	9.8	7.56	6.38	12.03	9.91	11.86	9.3
Unigene22805_CR24644-1S	**SeR1**	4.59	4.5	3.22	2.23	3.01	2.5	2.37	2.12	6.61	6.18	4.94	3.49	8.84	6.83	8.84	7.36
Unigene27183_CR24644-1S	**DoT**	1.66	1.19	1.06	0.68	1.8	1.64	0.79	0.83	2.39	2.69	2.04	1.56	3.88	3.21	3.51	2.45
Unigene2278_CR24644-1S	**DoR**	3.33	2.8	2.3	1.85	1.56	2.01	1.41	1.44	3.59	3.36	2.37	2.49	4	3.08	3.99	3.39
Unigene14920_CR24644-1S	**DoR1**	0.51	1.37	1.16	1.05	2.25	2.27	1.13	1.56	2.39	2.95	1.9	1.46	1.47	0.91	1.1	1.02
Unigene3902_CR24644-1S	**DoR2**	1.86	1.27	1.75	0.74	1.12	0.86	1	1.12	3.01	3.91	2.12	1.37	3.9	2.5	3.13	2.36
Unigene1578_CR24644-1S	**OcR**	3.28	3.6	1.96	1.88	1.69	1.76	1.47	1.49	4.46	3.48	2.52	2.28	4.14	3.03	4.36	3.39
Unigene26841_CR24644-1S	**OcR1**	17.03	18.63	11.92	10	11.83	9.53	8.77	8.56	22.85	21.33	16.88	15.7	18.41	14.66	20.57	17.47
Unigene78_CR24644-1S	**OcR2**	3.37	3.1	2.06	1.96	2.08	1.85	1.96	1.47	4.12	3.64	3.28	2.75	7.74	5.91	8.49	6.05
Unigene16540_CR24644-1S	**OcR3**	10.07	10.08	6.75	5.73	7.3	6	6.11	5.19	11.72	10.23	10.18	9.28	14.51	11.01	15.96	12.77
CL921.Contig1_CR24644-1S	**JHE**	2.7	2.82	1.77	5.42	2.44	4.38	2.74	5.92	4.7	2.37	1.71	1.7	14.97	13.39	12.2	9.16
Unigene1770_CR24644-1S	**JHEH**	20.7	21.98	24.4	16.79	20.87	17.1	20.43	19.97	20.78	20.51	26.93	17.03	22.16	29.92	19.74	16.73
CL2421.Contig1_CR24644-1S	**JHBP**	8.87	7.43	6.85	4.13	13	13.65	12.24	13.91	15.17	15.98	17.17	14.18	8.75	4.34	6.89	8.32
CL1764.Contig3_CR24644-1S	**JHAM**	11.14	13.9	12.07	13.51	24.24	23.07	18.88	17.55	21.92	23.13	19.17	18.33	14.51	14.61	11.91	11.4
Unigene27431_CR24644-1S	**EcR**	6.41	5.66	7.09	6.48	9.86	10.34	10.76	7.97	8.84	10.1	7.91	8.3	21.87	17.38	11.4	13.29
Unigene16583_CR24644-1S	**EcP74**	15.66	13.91	12.56	14.02	9.68	8.42	10.97	13.09	10.01	6.09	8.25	9.97	20.79	17.87	16.67	15.85
Unigene1807_CR24644-1S	**EcP75**	36.77	38.89	31.18	26.17	35.98	32.75	27.49	34.68	42.75	42.7	38.62	35.5	49.46	40.92	40.9	29.57
Unigene22062_CR24644-1S	**EcP78**	9.55	13.33	9.7	6.71	11.09	10.82	8.35	7.85	14.29	16.68	14.46	11.42	11.02	13.63	10.58	9.1
Unigene2986_CR24644-1S	**EcP93**	7.02	7.69	7.83	6.48	8.41	7.21	12.46	17.24	10.32	8.49	37.46	44.68	11.2	8.91	16.63	14.26
CL1523.Contig1_CR24644-1S	**InR**	2.07	2.47	1.44	1.37	2.81	3.12	2.23	2.6	3.14	3.45	3.11	3.21	10.22	8.64	8.15	5.32
Unigene1114_CR24644-1S	**IRS**	16.2	11.62	12.9	13.02	15.95	18.13	15.37	14.61	17.65	20.03	17.62	17.12	26.47	23.67	21.14	16.31

Table S6. FPKM of wing-associated genes among offspring in crowded and solitary conditions.

**Gene ID**	**Annotation Gene**	**CR24 644-1**	**SOL24 644-1**	**CR24 644-2**	**SOL24 644-2**	**CRAP2 4644-3**	**SOLAP2 4644-3**	**CRAL2 4644-3**	**SOLAL2 4644-3**	**CRAP2 4644-4**	**SOLAP2 4644-4**	**CRAL2 4644-4**	**SOLAL2 4644-4**	**CRAP2 4644-5**	**SOLAP2 4644-5**	**CRAL2 4644-5**	**SOLAL2 4644-5**
Unigene20375_CR24644-1S	***Fl***	3.39	0.44	0.23	0.1	0.32	0.46	0.43	1.87	4.64	4.73	193.61	239	285.54	10.77	2882.15	2503.72
Unigene1932_CR24644-1S	***Wg***	3.63	3.85	4.05	3.71	8.59	8.29	5.58	5.41	7.77	7.42	4.05	4.55	5.42	5.96	3.36	3.81
Unigene15256_CR24644-1S	***Dll***	9.94	12.47	8.71	10.89	31.7	31.43	19.22	20.11	30.11	28.52	21.28	21.1	29.58	30	20.78	19.43
Unigene1458_CR24644-1S	***En***	2.13	2.3	3.38	3.98	9.64	10.67	7.17	6.7	7.76	8.57	6.96	6.87	6.8	7.67	4.73	4.13
Unigene2552_CR24644-1S	***Vg***	2.94	3.43	3.14	2.19	5.09	4.59	6.47	6	5.8	6.06	13.35	9.31	5.71	4.71	5.37	4.38
Unigene1154_CR24644-1S	***Ap***	9.25	11.89	12.23	9.58	21.55	18.5	13.26	14.88	25.44	20.55	16.36	15.94	19.32	20.59	16.07	12.59
CL2052.Contig1_CR24644-1S	***Ubx***	3.06	3.12	2.61	2.69	10.76	11.28	4.87	5.2	9.82	10.5	8.78	7.16	11.89	11.96	9.41	7.85
Unigene4520_CR24644-1S	***Hh***	11.59	14.41	12.99	12.48	17.19	16.74	15.49	16.26	16.51	18.82	17.39	17.36	13.82	14.87	11.37	10.63
Unigene91_CR24644-1S	***Al***	0.28	0.33	0.16	0.43	2.13	2.44	1.03	0.98	1.53	1.64	1.44	1.43	4.1	5.12	2.45	2.16
Unigene1507_CR24644-1S	***Hth***	7.63	7.07	6.49	6.74	19.82	19.43	11.39	10.45	17.88	18.88	12.8	11.19	18.92	21.04	15.1	11.95
CL3016.Contig2_CR24644-1S	***Ser***	3.66	4.02	2.44	2.98	8.42	10.29	6.22	5.68	8.47	10.25	6.16	8.85	7.1	8.72	5.46	5.43
CL3031.Contig1_CR24644-1S	***Ptc***	1.07	2.63	1.67	1.89	4.8	4.49	4.11	3.18	3.57	5.27	3.95	3.42	8.78	8.25	5.32	3.98
Unigene1795_CR24644-1S	***Ci***	38.01	44.17	43.12	40.12	72.59	75.92	56.67	61.54	71.11	74.73	55.55	57.9	52.74	61.99	40.82	34.14
Unigene3083_CR24644-1S	***Brk***	5.26	6.03	4.93	4.82	14.05	13.94	9.92	9.23	12.15	13.29	11.48	10.51	13.15	12.6	10.49	9.16
Unigene16291_CR24644-1S	***Asc***	8.28	7.16	15.6	16.13	28.92	33.73	24.34	23.46	22.15	21.68	9.56	11.23	26.83	33.78	19.65	16.59
Unigene26859_CR24644-1S	***Srf***	4.73	4.26	4.15	2.56	7.18	6.21	5.17	6.69	8.01	8.33	24.32	18.84	12.42	12.9	11.88	10.14
Unigene2565_CR24644-1S	***Tsh***	8.89	9.18	8.49	7.86	17.89	19.8	11.24	10.71	17.51	20.3	13.32	12.9	16.73	16.39	12.92	10.83
CL2948.Contig2_CR24644-1S	***Rho***	5.16	6.8	7.06	4.87	6.16	7.47	5.97	5.95	7.44	4.81	8.55	5.25	7.05	5	5.48	6.84
CL2978.Contig1_CR24644-1S	***Omb***	0.35	0	0	0.8	1.06	3.17	1.08	0.9	0.69	2.06	0.91	0.73	2.82	3.6	2.44	2.72
Unigene1985_CR24644-1S	***Nub***	6.84	7.92	6.8	6.58	10.49	10.04	7.59	7.81	11.63	13.44	13.37	12.38	14.05	15.84	11	8.67
Unigene27785_CR24644-1S	***Spalt***	1.76	2.15	1.38	1	3.41	3.37	1.8	2.38	4.38	4.69	2.75	3.08	4.97	5.43	4.24	2.94

Unigene12218_CR24644-1S	***Cdc42***	38.42	43.7	37.2	38.83	67.25	66.09	51.24	46.88	62.53	68.18	57.93	51	80.84	78.06	65.07	57.89
Unigene1068_CR24644-1S	***Antp***	4.97	4.34	5.09	4.93	15.46	16.82	9.15	8.33	14.61	14.97	9.74	8.97	12.87	14.33	10.25	7.97
Unigene1254_CR24644-1S	***Fng***	9.51	11.85	8.79	7.27	14.62	10.21	12.5	11.28	14.19	16.07	11.79	11.68	9.82	11.48	7.77	7.06
Unigene11268_CR24644-1S	***Uba1***	15.62	16.22	16.09	15.53	30.99	30.92	22.3	20.13	25.92	27.22	20.57	18.42	43.24	45.7	32.46	29.13
Unigene1590_CR24644-1S	***Foxo***	12.74	10.24	7.53	10.08	9.52	8.93	7.95	7.44	10.49	11.1	12.33	6.95	34.31	24.7	28.33	23.23
CL3281.Contig1_CR24644-1S	***Scr***	11.91	15.52	11.78	13.68	27.81	27.16	15.69	16.09	26.05	27.75	14.5	15.88	18.01	20.13	13.37	11.3
Unigene7563_CR24644-1S	***Dpp***	6.12	6.12	4.11	3.91	11.18	10.51	6.16	5.23	10.52	11.63	7.66	8.22	16.03	14.14	12.47	10.68
Unigene3359_CR24644-1S	***Notch***	24.15	33.56	43.49	28.97	41.87	41.43	42.59	41.6	34.62	44.47	58.3	36.2	27.6	48.11	22.61	20.85
Unigene6303_CR24644-1S	***Wnt1***	2.79	3.49	3.49	2.71	6.47	6.91	3.28	3.38	7.73	7.74	6.47	5.24	5.98	5.8	5.69	5.12
Unigene13744_CR24644-1S	***Wnt2***	6.48	9.26	16.77	16.21	26.81	30.07	24.44	26.33	22.73	23.34	20.05	21.48	17.46	21.57	12.6	12.27
Unigene2662_CR24644-1S	***Wnt11***	2.96	4.15	2.24	2.08	2.71	1.85	2.61	2.35	3.26	2.88	2.45	3.56	3.79	4.87	3.97	3.11
CL1802.Contig2_CR24644-1S	***Wnt16***	2.64	2.34	1.79	2.75	4.35	3.58	2.16	3.33	5.15	5.04	3.86	2.94	6.66	4.7	8.37	6.3

Table S7. Information on all the samples of *R. padi* in the analyses.

Sample name	Developmental stage	Morph	Rearing condition	Biological replicates
CR24644-1	the first instar	wingless	crowded	CR24644-1F
	the first instar	wingless	crowded	CR24644-1S
	the first instar	wingless	crowded	CR24644-1T
SOL24644-1	the first instar	wingless	solitary	SOL24644-1F
	the first instar	wingless	solitary	SOL24644-1S
	the first instar	wingless	solitary	SOL24644-1T
CR24644-2	the second instar	wingless	crowded	CR24644-2F
	the second instar	wingless	crowded	CR24644-2S
	the second instar	wingless	crowded	CR24644-2T
SOL24644-2	the second instar	wingless	solitary	SOL24644-2F
	the second instar	wingless	solitary	SOL24644-2S
	the second instar	wingless	solitary	SOL24644-2T
CRAP24644-3	the third instar	wingless	crowded	CRAP24644-3F
	the third instar	wingless	crowded	CRAP24644-3S
	the third instar	wingless	crowded	CRAP24644-3T
SOLAP24644-3	the third instar	wingless	solitary	SOLAP24644-3F
	the third instar	wingless	solitary	SOLAP24644-3S
	the third instar	wingless	solitary	SOLAP24644-3T
CRAL24644-3	the third instar	winged	crowded	CRAL24644-3F
	the third instar	winged	crowded	CRAL24644-3S
	the third instar	winged	crowded	CRAL24644-3T
SOLAL24644-3	the third instar	winged	solitary	SOLAL24644-3F
	the third instar	winged	solitary	SOLAL24644-3S
	the third instar	winged	solitary	SOLAL24644-3T

CRAP24644-4	the fourth instar	wingless	crowded	CRAP24644-4F
	the fourth instar	wingless	crowded	CRAP24644-4S
	the fourth instar	wingless	crowded	CRAP24644-4T
SOLAP24644-4	the fourth instar	wingless	solitary	SOLAP24644-4F
	the fourth instar	wingless	solitary	SOLAP24644-4S
	the fourth instar	wingless	solitary	SOLAP24644-4T
CRAL24644-4	the fourth instar	winged	crowded	CRAL24644-4F
	the fourth instar	winged	crowded	CRAL24644-4S
	the fourth instar	winged	crowded	CRAL24644-4T
SOLAL24644-4	the fourth instar	winged	solitary	SOLAL24644-4F
	the fourth instar	winged	solitary	SOLAL24644-4S
	the fourth instar	winged	solitary	SOLAL24644-4T
CRAP24644-5	adult	wingless	crowded	CRAP24644-5F
	adult	wingless	crowded	CRAP24644-5S
	adult	wingless	crowded	CRAP24644-5T
SOLAP24644-5	adult	wingless	solitary	SOLAP24644-5F
	adult	wingless	solitary	SOLAP24644-5S
	adult	wingless	solitary	SOLAP24644-5T
CRAL24644-5	adult	winged	crowded	CRAL24644-5F
	adult	winged	crowded	CRAL24644-5S
	adult	winged	crowded	CRAL24644-5T
SOLAL24644-5	adult	winged	solitary	SOLAL24644-5F
	adult	winged	solitary	SOLAL24644-5S
	adult	winged	solitary	SOLAL24644-5T
